# Liberal and Sensible Construction of the Prohibition Law

**Published:** 1908-05

**Authors:** 


### LIBERAL AND SENSIBLE CONSTRUCTION OF THE PROHIBITION LAW.

The court of appeals rendered a sensible decision in reversing the construction of the lower court in regard to medicines, culinary and toilet articles. Undoubtedly this view will strengthen rather than weaken the present law, as it will increase the general respect for the law in that it carries out its intent and purpose. The court held that the intent of the legislature in enacting the law was to prevent the evils of intemperance caused by the use of intoxicating liquors as a beverage.

The court also held that the phrase "place of business," as used in that section of the act prohibiting the keeping of liquors in such places, meant a public place of business in contra-distinction to a place of private business.

In conclusion it says: "Prohibition is not a crusade against medicine, culinary or toilet articles, or those beverages which cannot intoxicate. These things are necessary to life, health and comfort. Prohibition is a crusade against intoxicating liquors as a beverage, and the resultant evils of intemperance."

